# Supplementary material for: Dynalign II: common secondary structure prediction for RNA homologs with domain insertions
Source: Nucleic Acids Res. 2014 Nov 21;42(22):13939–48. doi: 10.1093/nar/gku1172 (PMC4267632; doi:10.1093/nar/gku1172)
Supplement: SUPPLEMENTARY DATA [file supp_gku1172_nar-02021-z-2014-File012.zip › manual/Overview/AlphabeticalIndex.html]

RNAstructure Installation and Overview: Index


|  |  |  |
| --- | --- | --- |
|  | RNAstructure Installation and Overview Index | - Contents - Index |
| A B C D E F G H I J K L M N O P Q R S T U V W X Y Z --- A \| N; B Building Requirements Building Requirements: Compilers Building Requirements: Compiler Flags Building Requirements: CUDA Building Requirements: DATAPATH Variable Building Requirements: RNAstructure GUI Essential Software Building Requirements: RNAstructure GUI Essential Variables Building Requirements: Windows Interfaces Building the Command Line (Text) Interfaces Building the Command Line (Text) Interfaces for CUDA Building the GUI Interfaces Building the RNAstructure Repository Building SMP Parallel Programs \| O Object File Notes; C Components of the RNAstructure Repository \| P; D \| Q; E \| R Repository Organization Running the JAVA GUI; F \| S; G \| T; H \| U Update History; I Installation Instructions: Linux 32 and 64-bit Installation Instructions: Macintosh Installation Instructions: Windows Interface Summary: AllSub Interface Summary: bifold Interface Summary: bipartition Interface Summary: CircleCompare Interface Summary: ct2dot Interface Summary: dot2ct Interface Summary: draw Interface Summary: DuplexFold Interface Summary: dynalign Interface Summary: dynalign-smp Interface Summary: DynalignDotPlot Interface Summary: efn2 Interface Summary: EnergyPlot Interface Summary: EnsembleEnergy Interface Summary: Fold Interface Summary: JAVA Cross Platform GUI Interface Summary: MaxExpect Interface Summary: multilign Interface Summary: multilign-smp Interface Summary: NAPSS Interface Summary: oligoscreen Interface Summary: OligoWalk Interface Summary: partition Interface Summary: PARTS Interface Summary: ProbabilityPlot Interface Summary: ProbablePair Interface Summary: ProbKnot Interface Summary: refold Interface Summary: RemovePseudoknots Interface Summary: scorer Interface Summary: stochastic Interface Summary: TurboFold Interface Summary: TurboFold-smp Interface Summary: Windows Native GUI Interface Summary: Windows Text Interfaces \| V; J \| W; K \| X; L Libraries Built by the RNAstructure Repository Libraries Built by the RNAstructure Repository: Dynalign Libraries Built by the RNAstructure Repository: Dynalign SMP Libraries Built by the RNAstructure Repository: Hybrid RNA Libraries Built by the RNAstructure Repository: Multilign Libraries Built by the RNAstructure Repository: Multilign SMP Libraries Built by the RNAstructure Repository: Oligo Libraries Built by the RNAstructure Repository: PARTS Libraries Built by the RNAstructure Repository: Postscript Libraries Built by the RNAstructure Repository: RNA Libraries Built by the RNAstructure Repository: RNAstructure GUI Libraries Built by the RNAstructure Repository: TurboFold Libraries Built by the RNAstructure Repository: TurboFold SMP \| Y; M Makefiles Makefiles: Java Interface Makefiles: Main Repository Makefiles: NAPSS Makefiles: OligoWalk Makefiles: PARTS Makefiles: Postscript Library Makefiles: RNA Class Makefiles: Testing Framework \| Z | | |
| Visit The Mathews Lab RNAstructure Page for updates and latest information. | | |
